# A single nucleotide substitution in the coding region of Ogura male sterile gene, *orf138*, determines effectiveness of a fertility restorer gene, *Rfo*, in radish

**DOI:** 10.1007/s00438-021-01777-y

**Published:** 2021-03-26

**Authors:** Hiroshi Yamagishi, Megumi Jikuya, Kanako Okushiro, Ayako Hashimoto, Asumi Fukunaga, Mizuki Takenaka, Toru Terachi

**Affiliations:** 1 Faculty of Life Sciences, Kyoto Sangyo University, Kamigamo, Kita, Kyoto, 603-8555 Japan; 2 Research Center of Botany, Kyoto Sangyo University, Kamigamo, Kita , Kyoto, 603-8555 Japan; 3 Graduate School of Science, Kyoto University, Kitashirakawa-Oiwakecho, Sakyo-ku, Kyoto, 606-8502 Japan

**Keywords:** Fertility restorer gene, Ogura cytoplasmic male sterility, *Orf138*, ORF687, PPR-code, Radish

## Abstract

Cytoplasmic male sterility (CMS) observed in many plants leads defect in the production of functional pollen, while the expression of CMS is suppressed by a fertility restorer gene in the nuclear genome. Ogura CMS of radish is induced by a mitochondrial *orf138*, and a fertility restorer gene, *Rfo*, encodes a P-type PPR protein, ORF687, acting at the translational level. But, the exact function of ORF687 is still unclear. We found a Japanese variety showing male sterility even in the presence of *Rfo*. We examined the pollen fertility, *Rfo* expression, and *orf138* mRNA in progenies of this variety. The progeny with Type H *orf138* and *Rfo* showed male sterility when their *orf138* mRNA was unprocessed within the coding region. By contrast, all progeny with Type A *orf138* were fertile though *orf138* mRNA remained unprocessed in the coding region, demonstrating that ORF687 functions on Type A but not on Type H. In silico analysis suggested a specific binding site of ORF687 in the coding region, not the 5′ untranslated region estimated previously, of Type A. A single nucleotide substitution in the putative binding site diminishes affinity of ORF687 in Type H and is most likely the cause of the ineffectiveness of ORF687. Furthermore, fertility restoration by RNA processing at a novel site in some progeny plants indicated a new and the third fertility restorer gene, *Rfs*, for *orf138*. This study clarified that direct ORF687 binding to the coding region of *orf138* is essential for fertility restoration by *Rfo*.

**Supplementary Information:**

The online version contains supplementary material available at 10.1007/s00438-021-01777-y.

## Introduction

Cytoplasmic male sterility (CMS) that presents a defect in the production of functional pollen is spread in more than 140 plant species (Laser and Lersten 1972). The CMS genes are produced by recombination of the mitochondrial genome and consist of novel open reading frames (orfs) (Hanson and Bentolila 2004). The orfs are translated into novel mitochondrial proteins resulting failure to produce functional pollen (Chen and Liu 2014). CMS is suppressed by nuclear genes called fertility restorer genes (Rf genes). Recent studies revealed that a majority of the proteins encoded by Rf genes belongs to the pentatricopeptide repeat (PPR) family. The functions of PPR proteins involve RNA cleavage, RNA destabilization, or translational inhibition (Dahan and Mireau 2013). Thus, the CMS-Rf systems are very important to understand the interactions between the nuclear and mitochondrial genomes in plants. Among the systems, Ogura CMS of radish is one of the most deeply studied systems because the CMS gene, *orf138*, and an Rf gene, *Rfo*, were identified (Gaborieau et al. 2016). However, the exact functional relationship between the two genes is still unclear.

Besides the importance in the biological studies, the CMS trait has been the most effective genetic tool for plant breeding (F_1_ breeding) with the goal of hybrid vigor. CMS provides an efficient and stable F_1_ seed production system for plant breeding because it naturally avoids self-pollination of seed parent plants. Brassicaceae crops show marked hybrid vigor and F_1_ breeding using Ogura CMS, which was originally found in a Japanese radish (Ogura 1968), has been conducted worldwide not only in radishes but also in various *Brassica* crops including *Brassica oleracea, Brassica napus*, and *Brassica juncea*. As aforementioned, the causal gene of the Ogura CMS is a mitochondrial gene, *orf138* (Bonhomme et al. 1991), which is located at the 5′ region of *atp8* and expressed as a bicistronic transcript together with *atp8* (Bonhomme et al. 1992; Krishnasamy and Makaroff 1993).

We observed that *orf138* is distributed in more than 40% of Japanese wild radishes (Yamagishi and Terachi 1996). Sequence analyses of *orf138* in large numbers of wild and cultivated radishes revealed six nucleotide substitutions and one insertion/deletion in the coding region of the gene (Yamagishi and Terachi 2001). Using the combination patterns of the seven nucleotide-variations, *orf138* were classified into nine haplotypes, Type A–I. We inferred that Type B is the ancestral form of the *orf138* from the comparison of the nucleotide variations among the nine types (Yamagishi and Terachi 2001). The Type B *orf138* was found in a wild species, *Raphanus raphanistrum*, as a major type, and it is linked with male sterility (Yamagishi and Terachi 1997, 2001). On the other hand, the same Type B *orf138* has also been found in European natural populations of *R. raphanistrum*, but surprisingly, it did not induce male sterility (Giancola et al. 2007), suggesting that at least one fertility restorer gene (Rf gene) for Type B *orf138* is widely spread in European natural population.

The phenotypic expression of male sterility is controlled by combinations of mitochondrial CMS genes and Rf gene(s), encoded in the nuclear genome. An Rf gene for Ogura CMS was identified by three groups and named *Rfo* or *orf687* (Brown et al. 2003; Desloire et al. 2003; Koizuka et al. 2003). This gene (hereafter, *Rfo*) alters expression of *orf138* at a post-transcriptional level (Desloire et al. 2003) and either directly or indirectly lowers the amount of ORF138 protein (Koizuka et al. 2003). *Rfo* encodes a 687 amino acid pentatricopeptide repeat (PPR) protein referred to as ORF687. ORF687 is thought to interact with the *orf138* mRNA, or to act on the post-translational stability of the ORF138 protein, since the amount and sequence of *orf138* mRNA was not altered by the presence of ORF687 (Bellaoui et al. 1999; Uyttewaal et al. 2008). Although Uyttewaal et al. (2008) proposed that ORF687 could associate with the 5′ untranslated region (5′ UTR) of *orf138* mRNA, the function of ORF687 to prevent the translation is still unclear. While, Koizuka et al. (2003) observed four amino acid substitutions in the second and third repeats of the PPR domain between an *rfo* allele in male sterile lines and an *Rfo* allele in fertility-restored lines. They inferred that the two PPR repeats in ORF687 are essential for fertility restoration (Koizuka et al. 2003). Furthermore, Imai et al. (2002) found that the 118th amino acid in the PPR protein among the four substitutions plays a crucial role for fertility restoration.

Based on these findings, we surveyed the distribution of the *Rfo* allele in Japanese wild radishes. However, the frequency of the *Rfo* allele was unexpectedly low, with about 15% plants possessing this allele though more than 90% plants had one or more Rf gene(s) (Yasumoto et al. 2008). Therefore, we searched for additional Rf genes and found a novel gene that works in the processing of *orf138* mRNA in the Japanese wild radish population and named it *Rft* (Yasumoto et al. 2009), although the gene has not yet been cloned. By the product of *Rft*, *orf138* transcript lost part of the 5′ coding region before the 102nd–104th nucleotides from the start codon (Yasumoto et al. 2009).

Our results indicated that at least nine types of *orf138* are distributed in wild and cultivated radishes, and that two or more Rf genes are present in radishes. However, the correlations between the various types of *orf138* and the effect of multiple Rf genes have not been well studied. In this report, we surveyed the types of *orf138* and the distribution of *Rfo* and *Rft* in commercially available modern cultivars in Japan. We also determined the DNA sequences of *orf138* as well as *Rfo* genes in respective cultivars. Furthermore, expressions of these genes and male sterility phenotypes in progeny produced by cross-hybridizations among the cultivars were compared. The results revealed interesting relationships between the *orf138* types and the function of ORF687. The validity of ORF687 toward *orf138* is disrupted by a single nucleotide substitution in the coding region of *orf138* where ORF687 binds directly to the *orf138* mRNA. Our data also suggest that another Rf gene other than *Rfo* and *Rft* is present in radishes. The new Rf gene named *Rfs* processes *orf138* mRNA at a different position from that of *Rft*.

## Materials and methods

### Plant materials

In our preliminary study, we found a radish variety, ‘BK’, which shows male sterility and possesses *RfoRfo* genotype. We employed ‘BK’ and ‘MR’, which showed the *rforfo* genotype and male sterility, for cross hybridization. We also used two cultivars ‘MS-Gensuke’ (‘MS-G’ in this article) and ‘Soubaika Kansaitou’ (‘SK’ in this article) that possesses Type A and Type H *orf138*, respectively (Yamagishi and Terachi 2001). ‘MS-G’ is a male sterile variety that we use as a control of the Ogura CMS. ‘SK’ showed normal pollen fertility, and the fact suggested that this variety encodes at least one Rf gene. We crossed ‘BK’ and ‘SK’ first. Using one hybrid plant of the cross, (‘BK’ × ‘SK’), as a common pollen parent, cross hybridization was carried out with ‘MR’ or ‘MS-G’ as a female parent and the progenies were used for further analyses. The radish plants were grown in a glass house.

### DNA and RNA isolation

Total DNA was isolated from young leaves using a DNeasy Plant Mini Kit (QIAGEN, California, USA). Total RNA was isolated from flower buds (about 0.1 g) using an RNeasy Plant Mini Kit (QIAGEN).

### Examination of *Rfo* genotypes and the presence of *Rft* locus

To investigate whether the plants had a DNA sequence encoding functional ORF687 (*Rfo*) or had the *rforfo* genotype, which lacks the Rf function, we performed PCR–RFLP analysis with a primer pair and the restriction enzyme *Ssp*I as described (Yasumoto et al. 2008). The presence of *Rft* was identified using the STS marker, following the reported procedure (Yasumoto et al. 2009). All primers used in this experiment, including those used to examine *Rfo* and *Rft* are shown in Table S1.

### Sequencing of *orf138* and *Rfo*

PCR products containing an entire coding and the 5′/3′-flanking regions of *orf138* were purified using a QIAquick PCR purification Kit (QIAGEN) and directly sequenced using a CEQ 8000DNA sequencer (Beckman Coulter, California, USA). The DNA sequences of *Rfo* were determined similarly, using a PCR product amplified with a primer pair described in Yasumoto et al. (2009). The amino acid sequence of ORF687 was deduced based on the DNA sequence.

### Northern blot of *orf138* and qRT-PCR of *Rfo* in fertile and sterile progeny plants

Northern blot analysis for *orf138* mRNA was conducted using total RNA isolated from flower buds according to the reported method (Yasumoto et al. 2009). Total RNA was hybridized with the probe located over the entire coding region of *orf138* (Probe I in Yasumoto et al. 2009). To examine the expression of *Rfo*, quantitative RT-PCR (qRT-PCR) was carried out. To do this, total RNA was extracted from the flower buds of three fertile and three sterile plants among the progeny of ‘MR’ × (‘BK’ × ‘SK’). After cDNA was synthesized, quantitative PCR was carried out using KOD SYBR^®^ qPCR Mix (TOYOBO, Osaka, Japan) with 7500 Real Time PCR system (Applied Biosystems, Massachusetts, USA). *Actin1* was used as an internal control for RNA expression.

### Detection of the processing site in the *orf138* mRNA

Circular RT-PCR was conducted with the methods described in Perrin et al. (2004) and Giancola et al. (2007). Briefly, total RNA was incubated with T4 RNA ligase (New England Biolabs, MA, USA), and cDNA was synthesized. The region containing the junction of 5′ and 3′ extremities was amplified by RT-PCR. The circular RT-PCR products were cloned using the PGem^®^—TEasy Vector Systems (Promega, Madison, USA), and the sequences were determined. The primers used in the procedure are shown in Table S1.

### Prediction of target site of ORF687

To predict the potential binding sites for the ORF687 variants from ‘BK’, ‘SK’, and ‘MR’, we used the FIMO program in the MEME suite (http://meme-suite.org/tools/fimo). We generated a putative nucleotide binding motif for ORF687 using the identities of the amino acids at the 5th and last position of each PPR motif. These nucleotide preference scores (Figure S1) were used to predict the best potential binding site search for ORF687 in Type A or Type H of *orf138* mRNA sequences using the FIMO program. The predicted binding sites were ranked by *P* values calculated by FIMO.

## Results

### A male sterile radish variety ‘BK’, as well as a male fertile ‘SK’, has Type H *orf138*

**Fig. 1:**
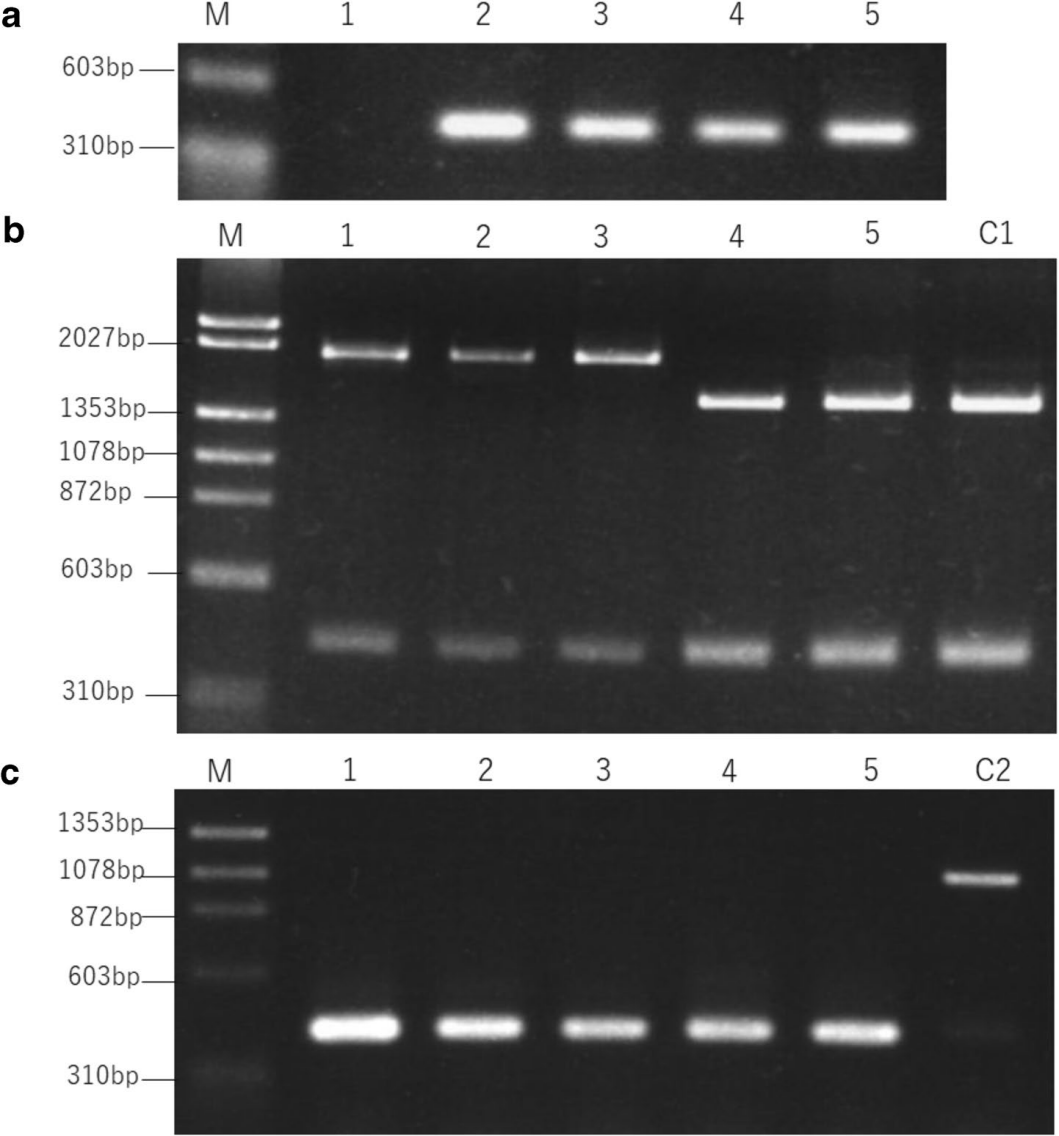
Presence of *orf138* and *Rft*, and genotyping of *Rfo* locus. **a** Detection of *orf138* DNA. **b** PCR–RFLP of *Rfo* locus to determine the genotype. **c** Detection of *Rft* by the STS marker. 1: ‘Uchiki-Gensuke’ (a maintainer of Ogura CMS). 2: ‘MS-G’. 3: ‘MR’. 4: ‘BK’. 5: ‘SK’. C1: A Japanese wild radish with *RfoRfo* genotype. C2: A Japanese wild radish having *Rft*

More than 10 male sterile Japanese radish varieties were observed by our preliminary survey and all of them possessed *orf138*. Although most of them had *rforfo* genotype at the *Rfo* locus, one of the male sterile cultivars named ‘BK’ possessed the *RfoRfo* genotype. We focused on ‘BK’ because this could provide a clue for understanding the molecular mechanism of *Rfo*-mediated fertility restoration. First, we ascertained that ‘BK’ and two other male sterile varieties, ‘MS-G’, ‘MR’, and a male fertile variety, ‘SK’, possess *orf138* by PCR (Fig. 1a). Next, the DNA sequences of all the coding and 5′/3′ flanking regions of *orf138* were determined for ‘MR’, ‘BK’, and ‘SK’. The three cultivars had Type H *orf138* (Table 1). In Type H, the 61st nucleotide from the AUG start codon of *orf138* is C whereas that in Type A (‘MS-G’) and Type B is A instead. The Type H has G at the 99th nucleotide of *orf138* as well as Type B, while Type A (‘MS-G’) has A (Yamagishi and Terachi 2001). Table 2 shows the nucleotide substitutions among the three haplotypes. Besides these two sites, the sequences of the *orf138* in ‘MR’ and ‘BK’ were identical to Type A (‘MS-G’), in correlation to our previous results (Yamagishi and Terachi 2001). Furthermore, Type A and Type H did not show any differences in the intergenic region between *trnfM* and *orf138* (data not shown).

**Table 1:** Type of *orf138*, genotypes and presence of Rf genes, and pollen fertility of radish cultivars

Cultivars	Type of *orf138*	Rf genes		Pollen fertility
		*Rfo* genotype	*Rft*	
MR	H	*rfo rfo*	–^a^	Sterile
BK	H	*Rfo Rfo*	–	Sterile
SK	H	*Rfo Rfo*	–	Fertile
MS-G	A	*rfo rfo*	–	Sterile

^a^– *Rft* is absent

**Table 2:** Nucleotide substitutions in the coding region of *orf138* among the three haplotypes

Haplotype	Nucleotide	Sites
	61	99
Type A	A	A
Type B	A	G
Type H	C	G

### Distribution of *Rfo* and *Rft* genes

A nucleotide variation inducing the substitution of the 118th amino acid of ORF687 was used as an RFLP marker (Yasumoto et al. 2008) and the genotypes of the *Rfo* locus were estimated. ‘MR’ and ‘MS-G’ showing male sterile phenotype and lacking Rf genes had the genotype of *rforfo* at the *Rfo* locus, while ‘SK’ with normal pollen fertility showed *RfoRfo* genotype as expected (Table 1, Fig. 1b). However, ‘BK’ showed the *RfoRfo* genotype despite that it exhibited the male sterile phenotype and had Type H *orf138,* as well as ‘MR’ (Table 1, Fig. 1b). In addition to the genotype at the *Rfo* locus, we examined the presence of the *Rft* locus with a STS marker. All the varieties analyzed here showed the DNA amplification pattern of one lacking *Rft* (Table 1, Fig. 1c), indicating that the fertility restoration of ‘SK’ was not by the product of *Rft*.

### Amino acid sequence of ORF687 was identical between ‘BK’ and ‘SK’

Unexpected male sterility in ‘BK’ could be due to additional amino acid substitutions in ORF687 other than the 118th amino acid. Thus, we analyzed the DNA sequence of *Rfo* genes. The predicted amino acid sequences of ORF687 were completely identical between ‘BK’ and ‘SK’ despite the difference in their phenotypes (Table 3). The two cultivars (‘MR’ and ‘MS-G’) that showed *rforfo* genotype for the RFLP site mentioned above commonly had four different sites from ‘BK’ and ‘SK’ including the 118th amino acid (Table 3).

**Table 3:** Amino acid substitutions in ORF687 among the four analyzed radish cultivars

Cultivars	Amino acid positions in ORF687			
	118	153	170	171
MR	T	N	N	L
BK	N	T	D	F
SK	N	T	D	F
MS-G	T	N	N	L

### Another Rf gene other than *Rfo* and *Rft* restored male fertility

**Fig. 2:**
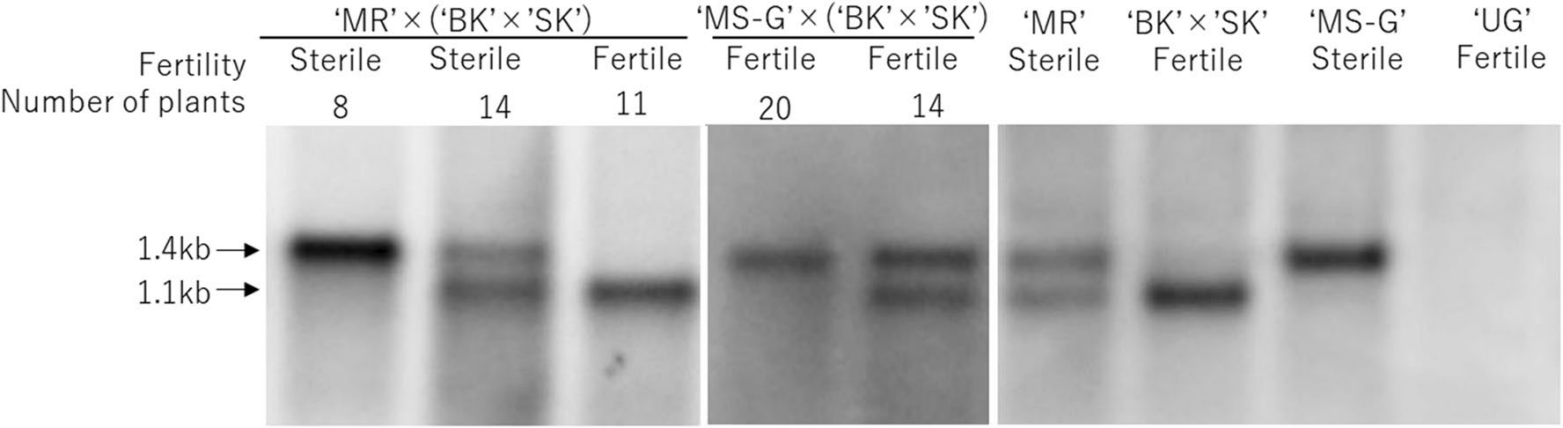
Transcript patterns of *orf138* and pollen fertility in the progenies of ‘MR’ × (‘BK’ × ‘SK’) and ‘MS-G’ × (‘BK’ × ‘SK’). ‘UG’; the maintainer of ‘MS-G’ (‘Uchiki-Gensuke’) used as a negative control

‘BK’ and ‘SK’ had identical characteristics in all aspects examined here except for pollen fertility; namely, the two varieties had Type H *orf138*, *RfoRfo* genotype, and lacked *Rft* (Table 1). To investigate the difference between the two varieties, we crossed them and hybrid plants were obtained. One of the hybrid plants, (‘BK’ × ‘SK’), was used to pollinate both ‘MR’ and ‘MS-G’. Among the characteristics we investigated, the only difference between ‘MR’ and ‘MS-G’ was the type of *orf138* (Table 1), ‘MR’ has Type H while ‘MS-G’ has Type A. The progeny of the cross ‘MR’ × (‘BK’ × ‘SK’) was segregated into 11 fertile and 22 sterile plants (Table 4). The *orf138* mRNA of the 11 fertility-restored progenies was processed in the coding region of *orf138*, showing about 1.1 kbp in size (Table 4, Fig. 2), even though they lacked *Rft*. In the sterile plants, the *orf138* mRNA was either partially processed (14 plants) or had the full-length *orf138* mRNA (8 plants) (Table 4, Fig. 2). This result indicated that the Type H *orf138* of ‘MR’ induced male sterility when the mRNA was not completely processed in the coding region, even though the genotype of the *Rfo* locus was *Rforfo* in all progeny plants*.* In contrast, all of the progeny plants from the cross ‘MS-G’ × (‘BK’ × ‘SK’) had normal pollen fertility (Table 5). Moreover, the *orf138* mRNA in these 34 progeny plants was either partially processed (14 plants) or not processed in the coding region (20 plants) (Table 5, Fig. 2). Pollen fertility was restored in all the progeny plants despite the fact that *orf138* mRNA was not perfectly processed in the coding region in the progeny of ‘MS-G’. The results indicated that the *Rfo* functions on Type A *orf138*. The expression of *orf138* in the presence of *Rfo* depended on whether the *orf138* was Type A or Type H. The expression of Type A *orf138* (‘MS-G’) was suppressed even though the mRNA was kept in full-length. On the other hand, Type H *orf138* (‘MR’) caused male sterility when the mRNA was not processed in the coding region.

**Table 4:** Pollen fertility and transcript pattern of *orf138* in the progeny of ‘MR’ × (‘BK’ × ‘SK’)

Pollen fertility	Transcript pattern			
	1.4-kb	1.4-kb/1.1-kb	1.1-kb	Total
Fertile	0	0	11	11
Sterile	8	14	0	22
Total	8	14	11	33

**Table 5:** Pollen fertility and transcript pattern of *orf138* in the progeny of ‘MS-G’ × (‘BK’ × ‘SK’)

Pollen fertility	Transcript pattern			
	1.4-kb	1.4-kb/1.1-kb	1.1-kb	Total
Fertile	20	14	0	34
Sterile	0	0	0	0
Total	20	14	0	34

### *Rfo* was expressed in both the fertile and sterile progeny plants

To verify that the ineffectiveness of *Rfo* in the male sterile plants with Type H was not due to repression of the *Rfo* gene, the transcription of *Rfo* gene was investigated in fertile and sterile plants in the progeny of the cross ‘MR’ × (‘BK’ × ‘SK’). The ratio of transcribed mRNA of *Rfo* gene to that of *actin1* analyzed by qRT-PCR demonstrated high amounts of *Rfo* transcription both in the fertile and the sterile progenies (Table 6). The transcription level in the sterile plants was higher than that in the fertile plants (126.7%) (Table 6), but the difference was not statistically significant (*t* = 0.385), indicating that *Rfo* was expressed in the sterile plants at levels similar to those observed in fertile plants.

**Table 6:** Transcription of *Rfo* in the fertile and sterile plants of the progeny of ‘MR’ × (‘BK × ’SK’)

Pollen fertility	Transcription relative to *actin1*^a^
Fertile (A)	6.15 ± 1.00^b^
Sterile (B)	7.79 ± 4.15^b^
Ratio (B/A)	126.7%

^a^Transcription is shown as a relative ratio to *actin1* used as an internal control for RNA expression

^b^The data are shown as mean ± SD of three plants

### Processing site of *orf138* mRNA

**Fig. 3:**
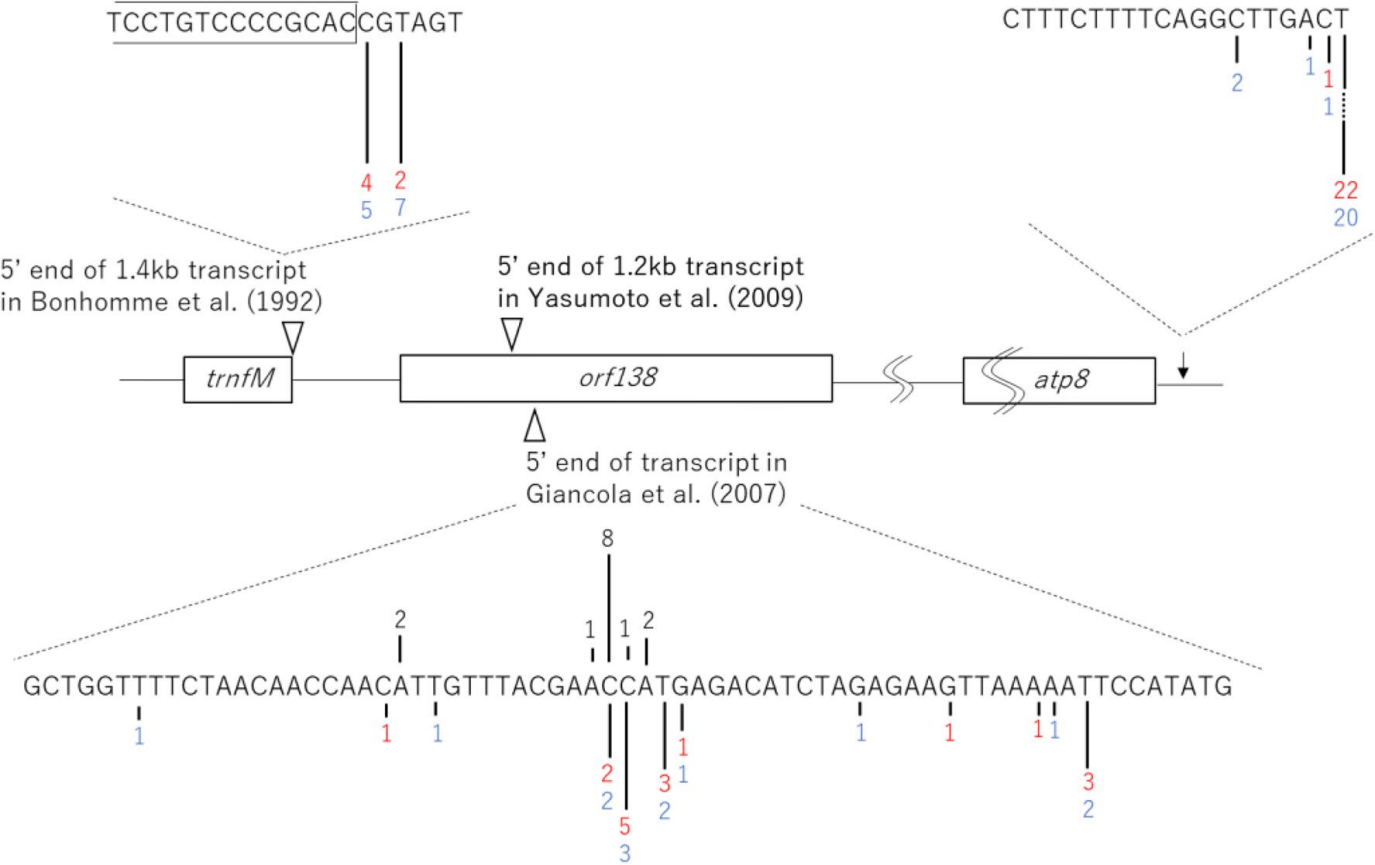
5′ and 3′ ends mapping of the *orf138* mRNA. Three arrow heads indicate the previously reported 5′ end in different radish varieties. The black arrow indicates the 3′ end of *orf138-atp8* co-transcript. Position of 5′ or 3′ ends and their frequency are indicated by bars and numbers, respectively. Red numbers (‘MR’ × (‘BK’ × ‘SK’), Blue numbers: ‘MS-G’ × (‘BK’ × ‘SK’). Black numbers: European wild radish (Giancola et al. 2007). The length of the bars reflects the frequency of each end

The Northern blot analysis demonstrated that all the fertile plants in the progeny of the cross ‘MR’ × (‘BK’ × ‘SK’) showed complete processing of *orf138* mRNA in the coding region (Table 4). Since the observed RNA processing was independent from the presence of the *Rft* gene, we conducted circular RT-PCR to determine the exact processing site in *orf138* mRNA. From 8 plants resulting from the cross ‘MR’ × (‘BK’ × ‘SK’), 4 of which were fertile and 4 were sterile, 24 clones were obtained. The majority of the clones (22 of 24 clones) had a 3′ end corresponding to the 3′ end of the *orf138-atp8* co-transcript irrespective of pollen fertility (Fig. 3). The precise 3′ ends of the clones was at the 120th nucleotide downstream of *atp8*; that is 8 nucleotides downstream of the 3′ end shown by Bonhomme et al. (1992), and 9 nucleotides upstream of that indicated by Bellaoui et al. (1999). The six clones obtained from the three sterile plants had 5′ ends at + 1 (four clones) or + 3 (two clones) downstream of the *trnfM* coding sequence and a full-length *orf138* mRNA (Fig. 3). The 5′ termini were identical to those previously reported (Bellaoui et al. 1999; Yasumoto et al. 2009). The other 8 clones derived from the sterile plants and nine clones from the fertile plants had 5′ ends ranging from the 143rd to the 183rd nucleotide of the coding sequence of *orf138*; the 5′ ends most frequently observed corresponded to the 156th (2 clones), the 157th (5 clones), and the 159th (3 clones) (Fig. 3). Although there was another clone with a 5′ end 17 bp upstream of the site processed by the *Rft* product, clones with the same 5′ end produced by the processing by *Rft* products (i.e. the 102nd–the 104th nucleotides) were not obtained. Thus, the majority of the *orf138* mRNAs derived from Type H (‘MR’) had either the full-length mRNA or a mRNA that was processed at around the 156th–159th nucleotide.

A total of 32 clones were obtained from the eight plants from the cross ‘MS-G’ × (‘BK’ × ‘SK’) that were all fertile. For 20 of these clones, the 3′ ends of the *orf138-atp8* co-transcript were at the same site (the 120th nucleotide) as was found for the progenies of the cross ‘MR’ × (‘BK’ × ‘SK’) (Fig. 3), and the 3′ end of four clones were placed 1–6 nucleotides further upstream of this position. The other 8 clones had the 3′ termini within the coding sequence of *atp8*. With regard to the 5′ end, none of these were completely processed in the coding region of *orf138* (Table 5). Twelve clones of the 32 had a 5′ end just downstream of *trnfM* (five clones at + 1 and seven clones at + 3) (Fig. 3). The 5′ end of 14 clones ranged from the 129th to the 183rd position from the start codon of *orf138*, and major sites were concentrated in the region containing the 156th (2 clones), 157th (3 clones), and 159th (2 clones) (Fig. 3). The other six clones had a 5′ end at 34–100 bp upstream of the *Rft* processing site (five clones) or far downstream of the major 5′ end sites (one clone). Again, no clones had a 5′ end at the resulting processed site by the product of *Rft*.

### The PPR-code suggested that ORF687 derived from *Rfo* allele prefers to bind to Type A *orf138* than to Type H

**Fig. 4:**
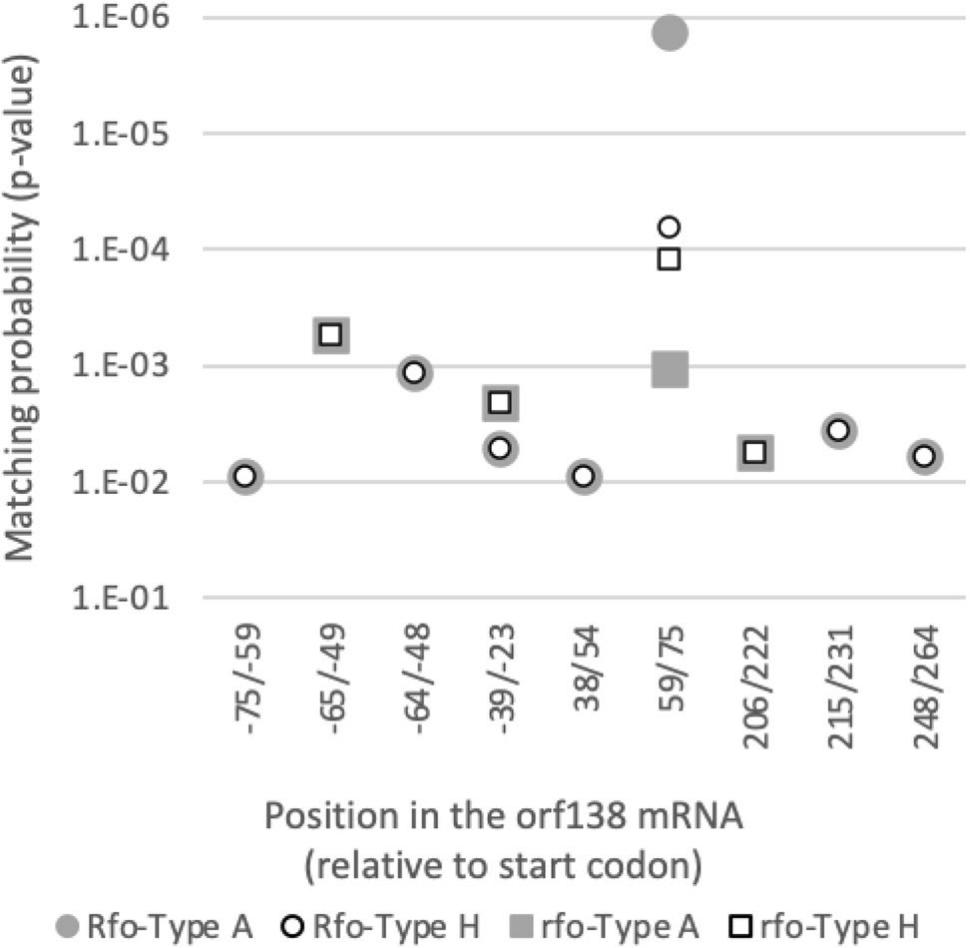
Putative binding sites of ORF687 in the orf138 mRNA were predicted based on the PPR-code. Matching probability to each putative binding site (*P* value) is blotted. ORF687 of ‘BK’ (Rfo) restores fertility to the Type A orf138 (gray circles), but not to the Type H (open circle). ORF687 of ‘MR’ (rfo) cannot restore fertility to either Type A (gray square) or Type H (white square)

Association of ORF687 to *orf138* mRNA in vivo was previously demonstrated (Uyttewaal et al. 2008), though its exact binding site has been unclear. The fact that the ORF687 from the *Rfo* allele functions only to Type A but not to Type H provided a hint for the exact binding site in the *orf138* mRNA. Therefore, we predicted the binding site of ORF687 derived from the *Rfo* allele in Type A or Type H *orf138* mRNA using the PPR-code (Yan et al. 2019). ORF687 contains 17 PPR domains (Table 7). Of these, 16 repeats were made up of 35 amino acids and one was 36 amino acids. We searched for the RNA sequence with which ORF687 from *Rfo* allele of ‘BK’ (hereafter called ORF687-BK) interacts with the highest possibility in the full-length transcript of Type A *orf138*. The top candidate of the binding sequences was 17 nucleotides (GUAAAGUUAGUGUAAUA) spanning from the 59th to the 75th position in the coding region of Type A *orf138* (Table 7, Fig. 4). Interestingly, this sequence contained a nucleotide substitution at the third position between the two *orf138* haplotypes, where Type A has A but Type H has C (Tables 2, 7). Target prediction of ORF687-BK toward Type H *orf138* also listed up the same 17 nucleotide sequence as the most probable binding site, though the affinity shown by the difference of P value decreased from 1.32E−06 with Type A to 6.51E−05 with Type H (Table 7, Fig. 4). This is because the threonine (T) and asparagine (N) at the two key positions for the PPR code in the third PPR motif of ORF687-BK corresponds exclusively to the nucleotide A, which Type A has but Type H does not. This is most likely the cause why ORF687 of ‘BK’ and ‘SK’ did not function to suppress the translation of Type H *orf138* mRNA derived from ‘MR’ when the transcripts were not processed in the coding region (Table 4).

**Table 7:** Alignment of the PPR codes of ORF687 and its putative binding site in the *orf138* mRNA

PPR no.	ORF687 of ‘BK’		*orf138* mRNA^a^		ORF687 of ‘MR’		*orf138* mRNA^a^	
	PPR code^b^ 5 × 35	Nucleotide preference	Type A	Type H	PPR code^b^ 5 × 35	Nucleotide preference	Type A	Type H
1	CD	G	G	G	CD	G	G	G
2	ND	U > C	U	U	TD	G > A	**U**	**U**
3	TN	A > G	A	**C**	NN	C > U	**A**	C
4	TT	A > G	A	A	TT	A > G	A	A
5	GN	A	A	A	GN	A	A	A
6	SD	G	G	G	SD	G	G	G
7	ND	U > C	U	U	ND	U > C	U	U
8	NN	C > U	*U*	*U*	NN	C > U	*U*	*U*
9	SN	A	A	A	SN	A	A	A
10	ND	U > C	**G**	**G**	ND	U > C	**G**	**G**
11	ND	U > C	U	U	ND	U > C	U	U
12	DD	–	G	G	DD	–	G	G
13	ND	U > C	U	U	ND	U > C	U	U
14	SN	A	A	A	SN	A	A	A
15	TN	A > G	A	A	TN	A > G	A	A
16	ID	–	U	U	ID	–	U	U
17	RF	?	A	A	RF	?	A	A
	*P* value		1.32e−06	6.51e−05	*P* value		1.04e−03	1.18e−04

^a^Normal letters indicate the matches between the PPR-code (nucleotide preference) for each PPR motif and corresponding nucleotide identity. Bold letters indicate the mismatches. Italic letters show the partial matches. The underlined letters indicate the PPR-code is neutral or unclear

^b^Amino acids with underline indicate the substitution between ‘BK’ (*RfoRfo*) and ‘MR’ (*rforfo*)

### Amino acid substitutions in ORF687 from *rfo* allele decreased affinity to *orf138* mRNA

The PPR code might also explain the distinct effectiveness between *Rfo* and *rfo* type ORF687. Thus, we compared the possible target sites of the two types of ORF687 between Type A and Type H *orf138*. The amino acid variations between the *Rfo* allele (‘BK’ and ‘SK’) and *rfo* allele (‘MR’ and ‘MS-G’) were located at the fifth amino acid of the second PPR (the 118th in Table 3) and the fifth of the third PPR (the 153rd in Table 3). Both of these two sites are crucial for the sequence specificity according to the PPR-code (Table 7), while the amino acid substitutions at the 170th and 171st positions (Table 3) corresponding to the 22nd and 23rd of the third domain are not likely to be involved in the nucleotide recognition. The amino acid substitutions in *rfo* changed the key amino acid residues for the PPR code from a N–D combination to T–D at the second motif and from T–N to N–N at the third motif (Table 7). The second repeat with a T–D combination could not recognize the second nucleotide (U), and the third repeat with a N–N combination much preferred the nucleotide C but not A in the Type A *orf138* (Table 7). Thus, these substitutions induced two and one mismatches with Type A and Type H, respectively (Table 7). Consequently, even if the ORF687 of ‘MR’ would bind the same 17 nucleotide region, the affinity to *orf138* mRNA was drastically lower than that of ‘BK’. The *P* values were 1.18E−04 with Type H and 1.04E−03 with Type A (Table 7, Fig. 4). These results clearly explain the reason that ‘MR’ and ‘MS-G’ are male sterile. The ORF687 product from *rfo* allele decreases affinity to the *orf138* mRNA due to the amino acid substitutions and could not suppress the translation of *orf138*.

In summary, our results strongly suggested that the ORF687 from ‘BK’ and ‘SK’ blocks the translation of Type A *orf138* mRNA by tightly binding to the mRNA in the coding region. However, the single nucleotide substitution from A in Type A to C in Type H at the 61st site lowered the binding affinity to the ORF687 and rendered the ORF687 ineffective for the suppression of *orf138*. On the other hand, the two amino acid substitutions in the ORF687 of ‘MR’ and ‘MS-G’ altered the PPR codes and diminished affinity to *orf138* mRNA. By these mutations, ‘MR’ and ‘MS-G’ having the *rforfo* genotype lost function as the restorer to Ogura CMS.

## Discussion

Here, we found evidence that the function of ORF687 protein, which is encoded by the *Rfo* gene, is to directly bind the mRNA of 17-nucleotides specific sequence in the coding region of *orf138*. The direct attachment of ORF687 to *orf138* mRNA disrupts the translation of *orf138*. Two amino acid substitutions at the 5th of the second and third PPR motifs alter sequence specificity of the ORF687 and determine the alleles to be either *Rfo* with the restoring ability or *rfo* which lacks the function. In the nucleotide sequence of *orf138*, where ORF687 interacts, one nucleotide substitution is present between Type A and Type H. Because of the substitution, the binding affinity of ORF687 to Type H transcript is significantly decreased, and ORF687 becomes ineffective. Our results also suggested that some cultivated radishes possess another Rf gene other than *Rfo* and *Rft*. The product of the new Rf gene named *Rfs* processes the *orf138* mRNA at around the 156th–159th nucleotide from the start of the coding sequence. It is of interest that Type A and Type H of *orf138* show different responses to *Rfs*, and further, that these responses are opposite of what was observed for their interactions with ORF687.

### Distribution of *orf138* haplotypes

We observed, in our preliminary survey, that more than 10 radish varieties sold in Japan showed male sterility and all of them had *orf138.* The *orf138* of most varieties were Type A, while a few including ‘MR’ and ‘BK’ used in this experiment had Type H *orf138*. The *orf138* whose sequence was published hitherto (Bonhomme et al. 1992; Krishnasamy and Makaroff 1993), and used worldwide for F_1_ breeding is Type A (Yamagishi and Terachi 2001). But Type A is not the major one in Japanese wild radishes and *R. raphanistrum*. Instead, Type B was mainly distributed both in Japanese wild radishes and *R. raphanistrum* (Yamagishi and Terachi 2001). Based on the distribution and the numbers of nucleotide variations among the types, we inferred that Type B is an ancestral form of *orf138* (Yamagishi and Terachi 2001). Type A *orf138* is different from Type B by a single synonymous change at the 99th site, and Type H has a single non-synonymous nucleotide substitution at the 61st site compared to Type B. The relationships between Type A *orf138* or Type H *orf138* and Rf genes were investigated in this article. It would be important to reveal the responses of Type B *orf138* to the Rf genes. In addition, though Type A was observed in Japanese wild radishes, Type H was unique to Taiwanese cultivars and was not found in wild radishes in our previous study (Yamagishi and Terachi 2001). Thus, the origin of Type H *orf138* of Taiwanese and Japanese cultivars remains to be studied further.

### Two amino acid substitutions in *rfo* allele affect the PPR code for ORF687

This is the first report to clarify the correlations between a nucleotide substitution in a mitochondrial CMS gene and the function of an Rf gene. The majority of Rf genes cloned to date encode proteins belonging to the P-type PPR protein family (Dahan and Mireau 2013). The P-type PPR proteins consist of 2–30 repeats of 35 amino acid length double helix motifs and associate with single strand RNA in a sequence specific manner. So far, annotated P-type PPR proteins participate in various aspects of organellar gene expression including transcription, RNA stabilization, 5′ processing, intron splicing, RNA editing, and translation (Barkan and Small 2014). The Rf gene (*Rfo*) of Ogura CMS encodes a member of the P-type PPR protein family (Brown et al. 2003; Desloire et al. 2003; Koizuka et al. 2003). Of the three reports, two of them, Brown et al. (2003) and Koizuka et al. (2003), described that the PPR encoded by the *Rfo* gene consists of 16 repeats, 15 comprised 35 amino acids and one comprised 36 amino acids. In the third study, Desloire et al. (2003) described that the PPR comprised 15 repeats of 35 amino acids, one of 36 amino acids and one with 46 amino acids. Later, Qin et al. (2014) found an additional PPR motif at the N-terminus using a web-based tool kit, TPRpred (Karpenahalli et al. 2007). In this report, we did not adopt the N-terminal sequence as a PPR motif, since recently published PPR motif prediction tools did not define it (Yan et al. 2019) (Table 7).

The amino acid substitution at the 118th position, which was postulated to have an important role for Rf function (Imai et al. 2002) is located at the 5th of the second motif. Interestingly, the 153rd amino acid, for which Imai et al. (2002) also found a substitution, was also present at the 5th position of the third motif (Table 7). These observations coincide with the prediction by Koizuka et al. (2003) that the second and third PPR motifs are essential for fertility restoration. PPR proteins function as sequence-specific single-stranded RNA binding proteins, and one RNA base coordinates with one PPR motif (Shen et al. 2015). To recognize a specific nucleotide, a combinatorial amino acid code in each PPR motif plays a key role (PPR-code). The key positions that confer RNA specificity were originally defined as the 6th amino acid of one repeat element and the first amino acid at the C-terminally adjacent repeat (6 and 1′) (Barkan et al. 2012; Barkan and Small 2014), while the same positions were annotated as 4 and ii by Yagi et al. (2013). Furthermore, following the structural analyses of PPR10, the same combinatorial di-residues were called the 5th and the 35th (Yan et al. 2019). These various numbering systems for the two crucial amino acid positions rely on the distinct definitions of a PPR unit in a PPR domain. Nevertheless, it is of much interest that both the 118th and the 153rd amino acid are placed at the 5th position (with the latest definition) of PPR motifs and function in nucleotide recognition (Table 7).

### Invalidation of ORF687 in ‘BK’ is due to neither the different sequence nor lower expression level of *Rfo* locus

The region containing the *Rfo* locus was described to consist of three PPR genes called *Ppr-A*, *Ppr-B* and a pseudogene *Ppr-C* (Desloire et al. 2003). PPR-A and PPR-B are composed of 686 and 687 amino acids, respectively, but the *Ppr-B* was assigned to the *Rfo* gene since only it acts as a fertility restorer gene. Crossing-over in the region around Rf loci has often made analysis of the Rf gene very difficult. For example, Wang et al. (2010) identified a chimeric gene that resulted from an inter-genic recombination between the two PPR loci. Such recombination created the rf type PPR gene even though the coding sequence for the PPR was identical to the Rf type. The rf type radish possessed the combination of a promoter region from a non-restoring allele and the coding region from the Rf type, and the PPR gene was not transcribed (Wang et al. 2017). Whereas in the Rf genes of petunia, that was the first cloned PPR protein coding gene, the difference between the fertility restoring genotype and the non-restoring genotype was in the promoter region, not in the coding sequence of the PPR protein (Bentolila et al. 2002). With this in mind, we compared the transcription of *Rfo* in fertile and sterile progeny plants; however, we found that *Rfo* was transcribed at equally high levels in both fertile and sterile plants (Table 6). This result allowed us to conclude that the reason for the failure of fertility restoration in the sterile plants resulting from the cross of ‘MR’ × (‘BK’ × ‘SK’) was not by the different transcription level of the *Rfo* gene.

### ORF687 directly binds the coding region of *orf138* to prevent translation

It has been already demonstrated that the accumulation of full-length *orf138* mRNA is not affected in the fertility-restored plants by ORF687. Thus, ORF687 must alter the expression of *orf138* at the post-transcriptional level. But the precise molecular mechanisms of the suppression have remained unknown. It had been described that the *orf138* transcripts were translated with the same efficiency in sterile and fertility-restored plants, and the ORF687 affected the post-translational stability of the ORF138 protein (Bellaoui et al. 1999). However, Uyttewaal et al. (2008) demonstrated that the primary role of ORF687 in restoring fertility was to inhibit ORF138 synthesis via its ability to directly or indirectly interact with the *orf138* RNA and postulated that the role of ORF687 was translational regulation of *orf138* mRNA. They proposed that ORF687 could associate with the 5′ UTR of *orf138* mRNA and prevented either the attachment or the progression of the mitochondrial translation machinery (Uyttewaal et al. 2008).

Our sequence analysis clarified that Type A and Type H *orf138* have identical 5′ processing sites as well as sequences in the 5′ UTR of *orf138.* Therefore, the association of ORF687 with the 5′ UTR of *orf138* mRNA proposed by Uyttewaal et al. (2008) cannot explain why ORF687 does not function to Type H *orf138*. Furthermore, our computational prediction strongly suggested that the association of ORF687 with *orf138* mRNA is present in the coding region. The 17 PPR motifs bind the 17 nucleotide-length target sequence in the mRNA, and the third position of the target sequence corresponds to the 61st nucleotide of *orf138* where Type A and Type H have A and C, respectively (Tables 2, 7). The 5th and 35th amino acid combination affecting the PPR code of the third motif is threonine and asparagine in ‘BK’ and ‘SK’, which strongly correlates with A (Shen et al. 2015). Therefore, the ORF687 strongly binds Type A *orf138*, which has A here, and consequently mitochondrial translational machinery somehow cannot function. In contrast, by the nucleotide substitution at the 61st site from A to C, the affinity of ORF687 to the target sequence is decreased in Type H, and the translation of *orf138* mRNA is not disturbed by ORF687. This scenario was further supported by the inactive ORF687 in the *rfo* allele. The second and third PPR motifs of the *Rfo* type ORF687 have high affinity to the 17 nucleotide targets in the Type A, while those in *rfo* type ORF687 have T–D and N–N combinations, respectively, and their nucleotides preference changes from UA to GC (Table 7). Accordingly, the *rfo* type ORF687 has less affinity to the 17 nucleotides target sequence in Type A as well as that in Type H *orf138* mRNA (Fig. 4). These genetic and PPR-code-based computational analyses strongly suggest that the function of ORF687 is to directly interact with *orf138* mRNA in the coding region and to suppress the translation of *orf138*.

### How does ORF687 suppress translation of ORF138?

Although our results strongly suggested that ORF687 directly binds to the coding region of *orf138*, it is unclear how the PPR–RNA interaction leads to suppression of translation. Several PPR proteins affecting translation in plant organelles have been reported (Manna 2015). Only a few such PPR proteins have been analyzed for their target sequences. PPR10 and PGR3 bind adjacent to the translation initiation site at *atpH* and *petL*, respectively, and resolve local stem-loop structures that would prevent the binding of ribosomes (Prikryl et al. 2011; Fujii et al. 2013). In contrast, ORF687 works as a negative regulator for translation. So far no PPR proteins which negatively regulate the mRNA specific translation have been described except for the ORF687. Secondary structure prediction of the *orf138* mRNA did not find a stem-loop including the start codon but found a stem-loop including another AUG, which is located 5 nucleotides upstream of the initiation codon. The stem-loop seems to suppress the translation from the upstream AUG and accordingly induces translation from the downstream AUG. If translation started from the upstream AUG, the translation would stop at a UAG overlapped with the initiation codon of the ORF138. Thus, translation from the downstream AUG would be suppressed. Binding of ORF687 to the 17-nucleotide target sequence in the coding region may rearrange secondary structure of the *orf138* mRNA and indirectly resolve the 5′ short stem-loop. It would be also possible that ORF687 work as a decoy for other RNA binding proteins, which bind around the translation initiation site and prevent translation of ORF138. Alternatively, stick binding of ORF687 to the target sequence might just physically impede moving of ribosomes at the site. In any case, further investigation will be necessary for understanding the molecular mechanism of translational suppression of ORF138 by ORF687. A PPR protein-mediated translational suppression is a new concept of gene regulation and will be another powerful tool to control expression of a given gene in plant organelles.

### A new Rf gene that processes *orf138* mRNA

In the progeny from the cross of ‘MR’ × (‘BK’ × ‘SK’), all of the fertile plants showed complete processing of the *orf138* mRNA in the coding region (Table 4). Many PPR proteins belonging to the restorer of fertility-like subfamily are often involved in 5′ end formation of mitochondrial transcripts in *Arabidopsis* (Jonietz et al. 2010; Stoll et al. 2014; Arnal et al. 2014). The *Rft* product also functioned in 5′ end formation (Yasumoto et al. 2009), but the processing sites were different from those observed in the progeny of ‘MR’ × (‘BK’ × ‘SK’). This finding indicates that ‘SK’ has a new and third Rf gene, different from *Rfo* and *Rft*. We named this Rf gene *Rfs*, and are planning to identify it. The processing sites by the product of *Rfs* varied clone-to-clone but were closely located to those observed for Type B *orf138* of the European wild radish (*R. raphanistrum*) (Giancola et al. 2007) (Fig. 3). The European Ogura related cytoplasm (Type B *orf138*) did not cause sterility, and it was interpreted to be due to cytoplasmic suppression of male sterility (Giancola et al. 2007). The similar processing sites at around the 156th–159th from the AUG initiation codon observed in the progeny of ‘MR’ × (‘BK’ × ‘SK’) strongly suggests that the European wild radish and ‘SK’ encode the same Rf gene. Unfortunately, Giancola et al. (2007) did not continue the project; however, the corresponding author of that study has suggested that their intriguing observation is attributable to one of the Rf loci present in wild radishes (Budar, personal communication). The new Rf gene, *Rfs*, found here has the ability to process the Type H *orf138* mRNA. However, it cannot process the Type A *orf138* mRNA of ‘MS-G’ (Table 5, Fig. 2). Type A *orf138* has A at the 99th nucleotide site where Type B and Type H have G (Table 2). It is necessary to clone *Rfs* and determine the amino acid sequence of the product in order to further understand the correlations between *Rfs* and the types of *orf138* at a molecular level.

## Supplementary Information

Below is the link to the electronic supplementary material.

Supplementary file1 (DOCX 15 kb)
