# Supplementary material for: A single nucleotide substitution in the coding region of Ogura male sterile gene, orf138, determines effectiveness of a fertility restorer gene, Rfo, in radish
Source: Mol Genet Genomics. 2021 Mar 26;296(3):705–17. doi: 10.1007/s00438-021-01777-y (PMC8144145; doi:10.1007/s00438-021-01777-y)
Supplement: Supplementary file 1 — Supplementary file1 (DOCX 15 kb) [file 438_2021_1777_MOESM1_ESM.docx]

**Electronic supplementary material**

**Table S1.** Primers used in this experiment

**Figure S1.** MEME Motif Format motif files for ORF687 in BK (ORF687_BK) and in MR (ORF687_MR). Each line indicates the expected nucleotide probability for each PPR motif. This motif file is available for FIMO program (http://meme-suite.org/tools/fimo).

**Table S1** Primers used in this experiment

| Usage | Forward(5’→3’) | Reverse(5’→3’) |
| --- | --- | --- |
| Detection of *orf138* | AATGATTACCTTTTTCGAAA | AATTTTCTCGGTCCATTTTCC |
| PCR-RFLP of *Rfo* | CGTTTATTTTCTCAGTCACGAG | CACTCTRTTCAACGATTTGA |
| Detection of *Rft* | GAACTCTGCAGTTACACAAGTC | AGTATAGTCCCTCAGAGTCTG  GAGAAGTAAAAACAATAAGATCC |
| Sequencing of *orf138* | CCTGTCGTTATCGACCTCGC | GCATGGGAGAAAACCAAGAAC |
| Sequencing of *Rfo* | ACAAGGAACTCAATCAATCAACTGG | GACATTGAAGCTCTGCTGCGC |
| qRT-PCR of *Rfo* | CCGGATCTTGTGATTTCTCTC | AGCTTAGAGCAGCTGCAGAA |
| qRT-PCR of actin1 | GTCGTACTACCGGTATTGTG | GTGCAAGTGCTGTGATCTCT |
| circularRT-PCR of *orf138* | CTACGGAACCAACTGCTTTC | CCCCTTTAATGATAGGTGAGAC |

MOTIF ORF687_BK

letter-probability matrix: length= 4 w= 17 nsites= 17

0.100000 0.100000 0.700000 0.100000

0.092371 0.247559 0.141649 0.518420

0.729074 0.049973 0.220952 0.000000

0.600000 0.050000 0.300000 0.050000

0.600000 0.300000 0.050000 0.050000

0.111111 0.092593 0.703704 0.092593

0.092371 0.247559 0.141649 0.518420

0.084860 0.554237 0.084500 0.276403

0.750000 0.100000 0.040000 0.110000

0.092371 0.247559 0.141649 0.518420

0.092371 0.247559 0.141649 0.518420

0.250000 0.250000 0.250000 0.250000

0.092371 0.247559 0.141649 0.518420

0.750000 0.100000 0.040000 0.110000

0.729074 0.049973 0.220952 0.000000

0.250000 0.250000 0.250000 0.250000

0.250000 0.250000 0.250000 0.250000

MOTIF ORF687_MR

letter-probability matrix: alength= 4 w= 17 nsites= 17

0.100000 0.100000 0.700000 0.100000

0.037078 0.000000 0.930407 0.032515

0.084860 0.554237 0.084500 0.276403

0.600000 0.050000 0.300000 0.050000

0.600000 0.300000 0.050000 0.050000

0.111111 0.092593 0.703704 0.092593

0.092371 0.247559 0.141649 0.518420

0.084860 0.554237 0.084500 0.276403

0.750000 0.100000 0.040000 0.110000

0.092371 0.247559 0.141649 0.518420

0.092371 0.247559 0.141649 0.518420

0.250000 0.250000 0.250000 0.250000

0.092371 0.247559 0.141649 0.518420

0.750000 0.100000 0.040000 0.110000

0.729074 0.049973 0.220952 0.000000

0.250000 0.250000 0.250000 0.250000

0.250000 0.250000 0.250000 0.250000

**Figure S1** MEME Motif Format motif files for ORF687 in BK (ORF687_BK) and in MR (ORF687_MR). Each line indicates the expected nucleotide probability for each PPR motif. This motif file is available for FIMO program (http://meme-suite.org/tools/fimo).
